# Performance of Melatonin as Prophylaxis in Geriatric Patients with Multifactorial Risk for Postoperative Delirium Development: A Randomized Comparative Study

**DOI:** 10.5152/TJAR.2022.20017

**Published:** 2022-06-01

**Authors:** Sherif Abdullah Mohamed, Ashraf Rady, Mona Youssry, Mennatallah Reda Abdelaziz Mohamed, Medhat Gamal

**Affiliations:** 1 Department of Anaesthesia, Cairo University Faculty of Medicine, Cairo, Egypt; 2 Department of Internal Medicine, Cairo University Faculty of Medicine, Cairo, Egypt; 3 Resident of Department of Anaesthesia, Cairo University Faculty of Medicine, Cairo, Egypt

**Keywords:** Abbreviated mental test, general anaesthesia, geriatric, melatonin, postoperative delirium

## Abstract

**Objective:**

Postoperative delirium is the worst patient outcome. Elderly patients undergoing orthopaedic procedures under general anaesthesia are highly liable to experience delirium. Several studies supported melatonin use for the prevention of delirium. This work evaluated the prophylactic efficiency of melatonin for postoperative delirium in patients with multifactorial risk for developing delirium as elderly undergoing orthopaedic trauma surgery under general anaesthesia.

**Methods:**

This double-blinded prospective randomized comparative study was conducted on 80 elderly patients subjected to orthopaedic interventions under general anaesthesia. Patients were randomized into group M (Melatonin group) and group NM (Non-melatonin group). Group M received 5 mg melatonin while group NM received placebo. The study drugs were given preoperatively and for the first 3 postoperative days. For the incidence of delirium, patients were evaluated using the Abbreviated Mental Test. The Pain Assessment in Advanced Dementia, sedation scores, and changes in hemodynamics were recorded.

**Results:**

The incidence of delirium was significantly lower postoperatively in M group (25%) relative to NM group (52.5%) (*P* < .001, OR = 2.3. 95% CI = −0.44, + 1.23). Abbreviated Mental Test scores at postanaesthesia care unit and day 0 showed a highly significant differences. However, Abbreviated Mental Test scores had no significant difference within the 3 postoperative days. Heart rate was significantly lower in M group after 50 minutes from the start of surgery. Mean blood pressure, Pain Assessment in Advanced Dementia, and sedation scores showed insignificant differences.

**Conclusion:**

Perioperative melatonin treatment could reduce the incidence of postoperative delirium in the studied population, and it could be considered a prophylactic medication.

## Main Points

- Prophylactic melatonin reduces postoperative delirium in geriatrics with multifactorial risk.
- The Abbreviated Mental Test was higher with melatonin.

## Introduction

Delirium is a condition characterized by disturbed consciousness and cognitive function changes including diminished concentration, unorganized thinking, abnormal psychomotor behavior, and irregular sleep-wake cycle.^1^ Postoperative delirium (POD) is expected to occur in 10-61% of elderly persons aging 65 years or older. A mortality rate of 10%-75% is likely to be linked to delirium once it develops, which may be in proportion to the age.^2^

A variety of precipitating factors of POD have been reported: age,^[Bibr b3-tjar-50-3-178]^ dementia, reduced myocardial function, electrolyte disturbance, alcoholism, smoking, increased blood transfusion needs, fluctuation of intraoperative blood pressure, and benzodiazepine usage.^3-5^ POD occurs frequently in certain procedures, such as orthopaedic operations,^6^ major gastrointestinal and major cardiovascular surgeries,^7,8^ surgery under general anaesthesia, prolonged surgery, trauma, and emergency surgeries.^9,10^ Also opioids have a variable effect on delirium occurrence with different agents.^11^

Various medications such as alpha 2-receptor agonists, atypical antipsychotics, and sleep regulators have been considered for the prevention or treatment of delirium, but their serious side effects may hinder their use.^12^

It was hypothesized that melatonin, a pineal gland-generated hormone, has a curative impact on delirium after surgery. It was effective, as a premedication, in sedation with a favorable cognitive profile.^13,14^

Several investigations in the old adults provided support for melatonin usage as prophylaxis in delirium or as a treatment. These studies involved different patient populations either in ICU, medical wards, or in surgeries.^15-18^

However, in this study, the chosen patients had multiple aggravating factors for the development of POD. These aggravating factors included old age, trauma, receiving opioids to alleviate the perioperative pain, orthopaedic surgery, and receiving general anaesthesia (GA). This double-blinded RCT aimed to investigate the preventive impact of melatonin against POD in this risky population. As to our knowledge, no adequate trials have been performed to evaluate the prophylactic ability of melatonin to mitigate the occurrence of postoperative delirium in patients with these combined challenging characteristics.

## Methods

This randomized, prospective, comparative, double-blind study was executed between 30 July 2020 and 15 December 2020 in the orthopaedic surgical units at Cairo University Hospitals. The study enrolled 80 geriatric patients undergoing orthopaedic trauma surgery under GA. The research protocol was accepted by the ethics committee of the Faculty of Medicine of Cairo University (ID: MS-103-2020). Written informed consent was signed by the patients participating in the research. The protocol was recorded at Clinicaltrial.gov (ID: NCT04483596 on 20/7/2020).

Inclusion criteria: Operations done in the morning, upper limb (accompanied or not with lower limb) orthopaedic trauma, receiving perioperative opioids to alleviate trauma pain, surgeries under GA, age 65 years old or more, both genders, and patients with the American Society of Anesthesiology (ASA) physical status I-III.

Exclusion criteria: Refusal of the patients, age less than 65 years old, ASA physical status ≥IV, allergy to the study medications, patients with Abbreviated Mental Test (AMT) score of <8, illiterates, preoperative sedation score >3, alcohol abuse, lost vision or hearing, hematocrit < 27%, cerebral insults (stroke, hemorrhage, infection), fluids and electrolyte abnormalities, acute cardiac problems (infarction, heart failure, dysrhythmias), acute respiratory events (asthma or chronic obstructive lung disease exacerbation, pulmonary embolism, hypoxemia, hypercarbia), drugs (anticonvulsants, antidepressants, antihistamines, antiparkinsonism agents, antipsychotics, melatonin), history of chronic sedative-hypnotic use > 3 times per week during a month before the surgery.

Patients were randomized equally into group M (melatonin group) and group NM (non-melatonin group) via computer-generated random numbers with sealed envelopes.

Preoperative management: All patients were requested to fast according to the standard rules. Every patient was assessed for relevant history and routine laboratory investigations, by an anaesthesiologist, on the night before surgery and screened for delirium at that time with the AMT (Table 1).^19^ Patients with an AMT score < 8 were assumed to have delirium. The Royal College of Physicians and the British Geriatric Society have recommended AMT examination for regular evaluation of the cognitive functions in older persons.^20^

Group M was provided a 5 mg tablet of melatonin, packed in an opaque envelope, orally at 9 in the evening before surgery and another 5 mg of melatonin with 15 mL of clear water 30 minutes before surgery. Group NM was provided a 500 mg tablet of paracetamol, as a placebo, as it looks similar to melatonin tablets, packed in the same way as melatonin at 9 in the evening before surgery and another 500 mg of paracetamol with 15 mL of clear water 30 minutes before surgery.

Upon arrival to the preparation area, a large-bore IV cannula was implanted into the nondominant side, and sedation was evaluated by Ramsay’s sedation score:^21^ Patients with a score > 4 were removed from the study. Heart rate (HR), systolic blood pressure (SBP), diastolic blood pressure (DBP), mean blood pressure (MAP), and oxygen saturation (SPO_2_) were recorded as the baseline readings.

The anaesthesiologist who gave the drugs and evaluated the AMT and sedation scores was blinded to the groups.

Intraoperative management: All participants were monitored with 5 lead ECG, noninvasive BP, pulse oximeter, capnography. HR, SBP, DBP, MAP, and SPO_2_ were recorded every 10 minutes. Induction of GA was done with fentanyl (2 μg kg^−1^), propofol (1-2 mg kg^−1^), atracurium (0.5 mg kg^−1^). Intubation was achieved with an oral endotracheal tube. Anaesthesia was maintained with isoflurane (1.15%), incremental 0.01 mg kg^−1^ atracurium for every 30 minutes. A 0.5 μg kg^−1^ fentanyl IV was given if HR and/or BP increased by 20% or more from baseline in response to surgical stimulation with a maximum of 4 μg kg^−1^.

Surgical duration, high perioperative transfusion needs (≥ 3 blood units), intraoperative fluctuation of pressure (< or > 30% of the baseline), and hypoxia (SPO_2_ < 92% on room air) were documented.

Postoperative management: HR, SBP, DBP, MAP, and SPO_2_ were recorded every 10 minutes until 30 minutes after extubation. Group M: 5 mg of melatonin was given at 9 PM on the day of the procedure and for the first 3 days.^15^ Group NM was provided a 500 mg paracetamol tablet at the same time as melatonin tablets in group M.

AMT scores were documented after recovery from postanaesthesia care unit (PACU AMT), 6 hours after surgery (Day-0) and on the next 3 postoperative days (Day-1, Day-2, and Day-3) at the same time of evaluation on day 0. Patients having a score < 8 were assumed to have POD^15^ and were advised to have further evaluation by a psychiatrist.

Postoperative pain was managed by IV 1 g of paracetamol every 8 hours, with an IV nalbuphine, as rescue analgesia, of 0.25 mg kg^-1^ not exceeding 0.5 mg kg^-1^ every 6 hours to maintain the Pain Assessment in Advanced Dementia scale (PAINAD) < 4 (Table 2). PAINAD is tested at times 0, 30 minutes 2, 4, 8, 12, 18, and 24 hours. The “zero” time was the moment of recovery from GA.^22^

Primary outcome was the detection of the difference in the incidence of POD between both groups at day 3 postoperatively. Secondary outcomes were targeting the between-group variables associ­ated with POD, Ramsay’s sedation scale, HR, SBP, DBP, MAP, and SpO_2_ from baseline to 30 minutes post-extubation and the PAINAD scale.

### Statistical Analysis

Using the Clincal.com sample size calculator, the sample size was calculated as 40 patients in each group by achieving 80% power with a significant alpha level of 0.05 and based on data from a pilot study which revealed that melatonin reduced the incidence of POD from 53.34% to 23.4%. Data analysis was done by using version 21.0 of the Statistical Package for Social Sciences (SPSS) (IBM Corp.; Armonk, NY, USA). Quantitative statistics were expressed as mean ± standard deviation (SD). Qualitative statistics were expressed as the number and percent of patients. The means of the groups were compared using the independent samples *t*-test. Evaluation of the proportions of qualitative parameters was done by the chi-squared test. Statistical significance was concluded when *P* value was less than .05.

## Results

**Figure 1.**
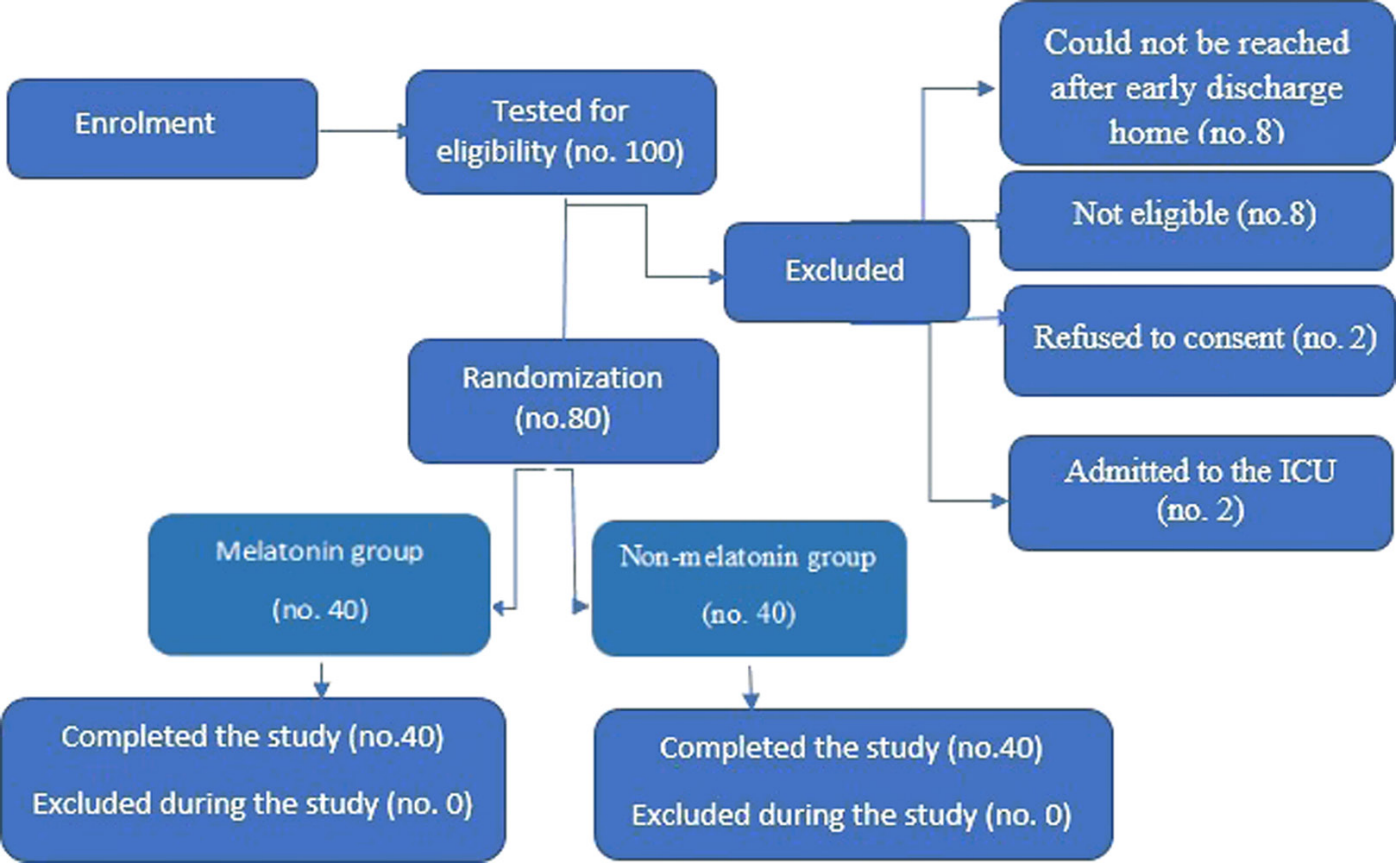
Flow chart for patients’ enrollment.

A total of 100 patients were enrolled, while 80 completed the study (Figure 1). Patients’ characteristics for the 80 patients involved are detailed in Table 3. A highly statistically significant difference was shown between groups as regards the incidence of POD with a significantly less incidence in group M (*P* < .001, OR = 2.3, 95% CI = -0.44, + 1.23) (Table 3). The risk variables comprising the surgical duration, high perioperative transfusion needs, intraoperative fluctuation of pressure, hypoxia, age, gender, BMI, ASA score showed no statistically significant difference between the groups (*P* > .05).

As regards AMT comparison between M and NM groups, the day before surgery exhibited no significant difference; however, it exhibited a significant difference at PACU (*P * = .001) and day [0] (*P * = .035). No significant statistical difference was found between groups during days [1] to [3], despite the relative superiority of M group over NM group. PAINAD score on the first postoperative day showed a nonsignificant between-group difference. Also, the incidence of POD was statistically significant on the postoperative days [0, 1] (Table 4).

**Figure 2.**
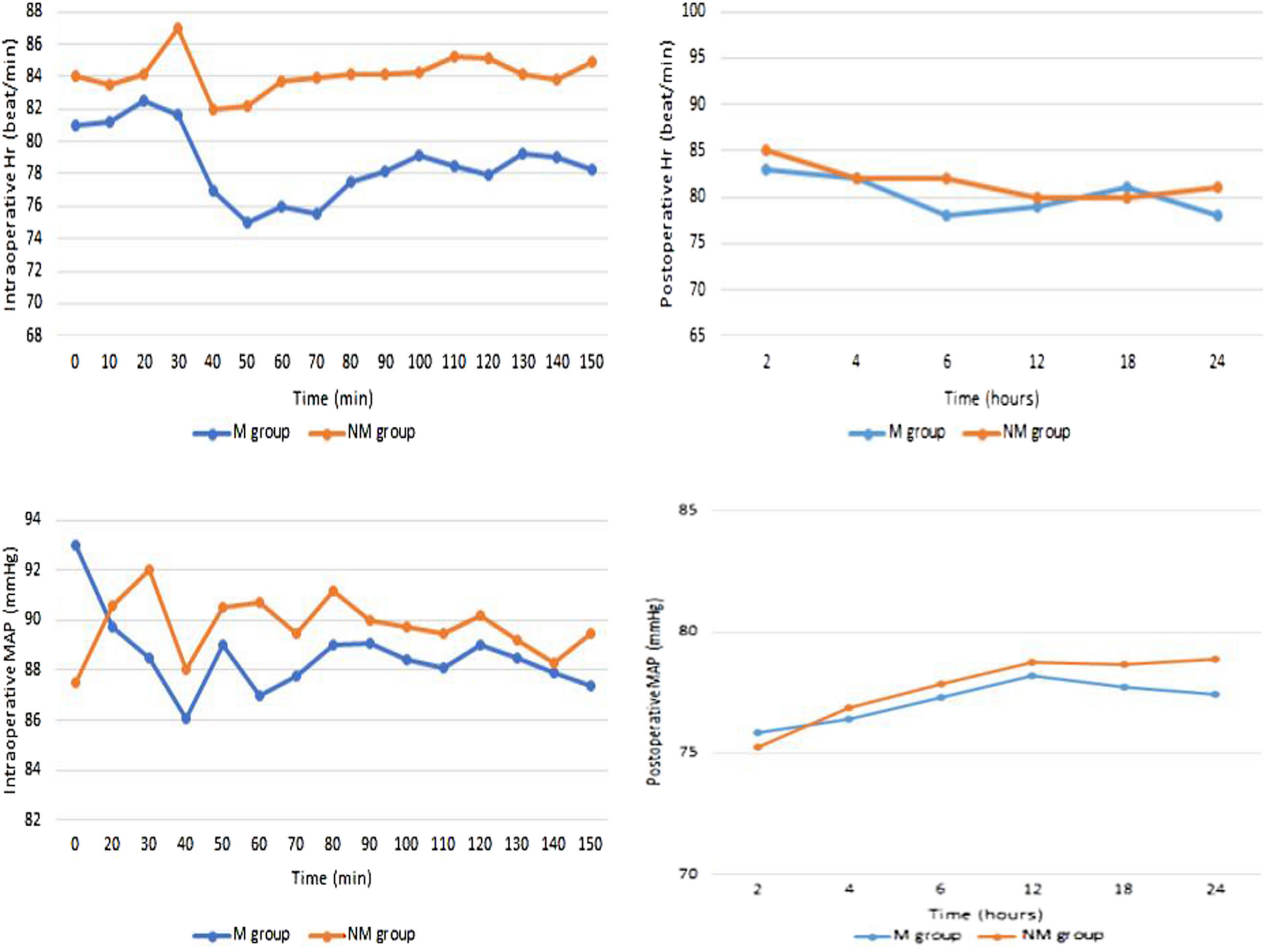
HR and MAP trends in M and NM groups. HR, Heart rate (beat per minute); MAP, Mean arterial pressure (mm Hg).

HR showed an insignificant difference between M and NM groups from baseline to 50 minutes followed by a period with a significantly lower HR in M group until the end of surgery. Postoperatively, there was an insignificant postoperative between-group difference for HR with F(1) = 1.7 and *P* = .22 (Figure 2).

**Figure 3.**
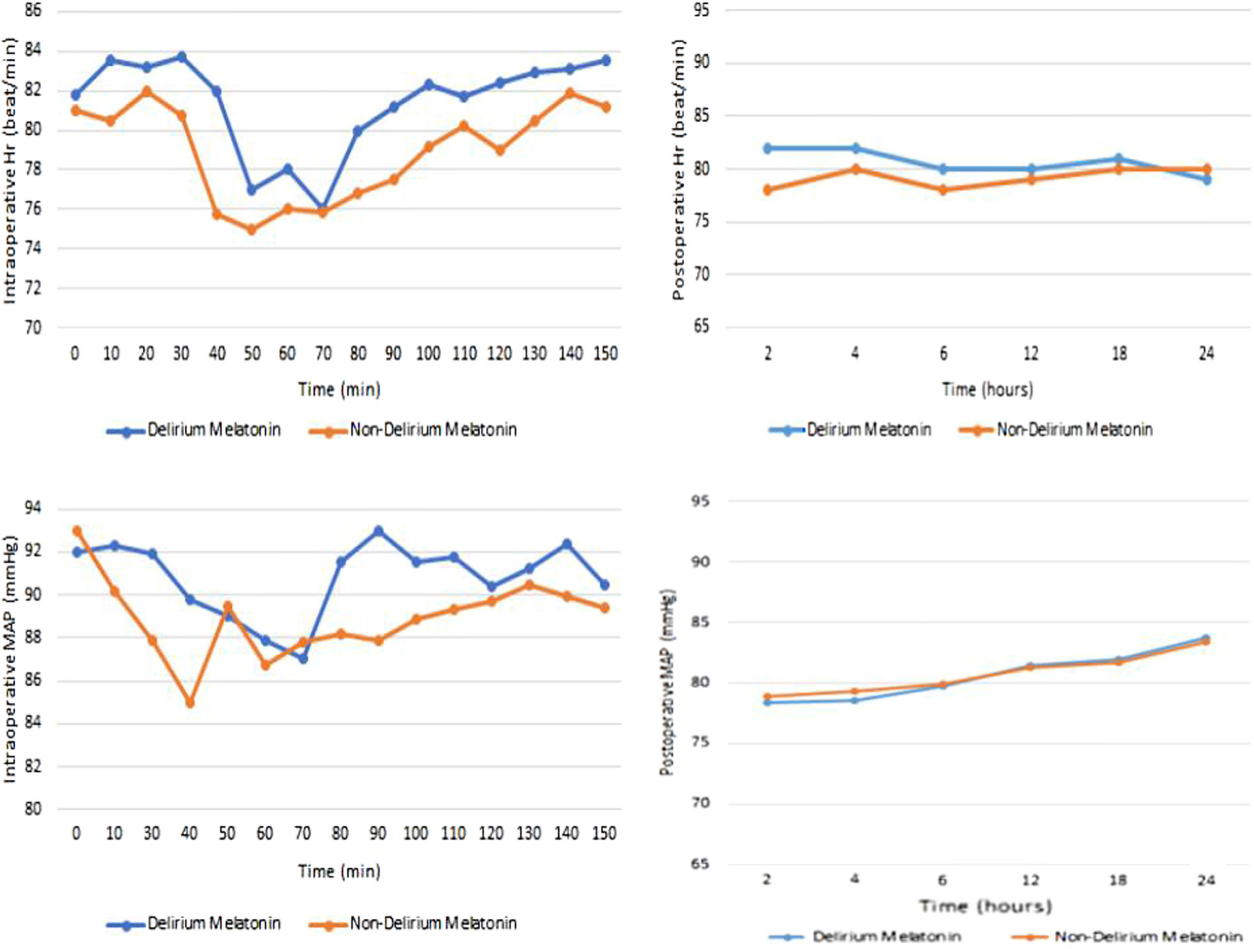
HR and MAP trends in delirium and non-delirium melatonin groups. Mean HR, Heart rate (beat per minute); MAP, Mean arterial pressure (mm Hg).

As regards MAP, the preoperative baseline values were significantly higher in M group while there was an insignificant intraoperative difference and insignificant postoperative difference with F(1) = 2.9 and *P* = .099 (Figure 3).

Ramsay’s sedation score fluctuated between 1 and 2 in both groups with an insignificant difference in the prevalence of either score in both groups (Table 3). Only 4 patients in each group were desaturated (92%-95%) in the first 30 minutes postoperatively and needed oxygen mask to maintain SPO_2_ (98%-100%). Insignificant difference in oxygen saturation existed between groups M and NM.

The comparison between patients who had delirium (**delirium melatonin group)** and patients who did not have delirium (**non-delirium melatonin**) group in patients in M group revealed a statistically significant difference as regards sedation score, BMI, and duration of surgery. The **non-delirium melatonin** group had a larger percentage of patients with a score of 2, while the **delirium melatonin group** had a higher BMI and a longer duration of surgery (Table 5). While there was an insignificant difference as regards HR and MAP (Figure 3).

**Figure 4.**
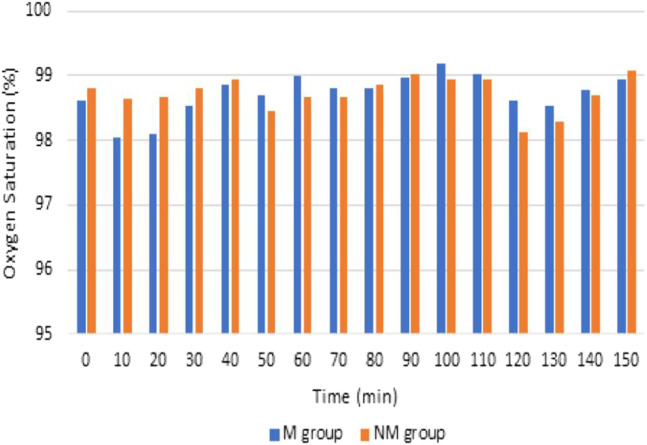
SPO_2_ trends in M and NM groups. Mean SPO_2 _(%) versus time in minutes.

As regards AMT score, there was a statistically significant difference between the delirium and non-delirium melatonin groups at PACU and day [0], with an insignificant difference in the rest of the recording period. As regards the PAINAD scale, there was no significant difference between the compared groups (Table 6). Also, there was no significant statistical difference between groups as regards to oxygen saturation (Figure 4).

## Discussion

This double-blinded randomized comparative clinical trial intended to explore the effectiveness of melatonin to decrease the incidence of POD in patients with several predisposing risk factors. The risky sample group of the study involved elderly patients with orthopaedic trauma operations under GA. Besides, patients were delivered opioids to manage their perioperative pain. Although melatonin was used in many studies to prevent or control delirium in vulnerable patients for POD, not enough previous researches had explored the prophylactic efficiency of melatonin against this particular challenging combination that exists in this study.^23,24^

The hypothesis was that melatonin will cause a significant drop in the incidence of POD in the chosen population. The results supported this theory, which revealed a highly significant difference between melatonin and non-melatonin in the percent of patients who had POD with a significantly less incidence in M group (25%) relative to the NM group (52.5%). The PACU and day [0] AMT scores revealed a significantly higher score in M group, but the next 3 days had no statistically significant difference between groups.

The higher incidence of delirium in the non-melatonin group relative to the melatonin group couldn’t be explained by the presence of risk variables (the surgical duration, high perioperative transfusion needs, intraoperative fluctuation of pressure, hypoxia, age, gender, BMI, and ASA score), as they showed no statistically significant intergroup difference. This means that both groups were subjected to similar conditions.

The study used paracetamol tablets as a placebo in the NM group, as they look similar to melatonin tables, in a dose of 500 mg and only once daily. This dose was hypothesized not to affect pain control significantly as it was reported to be less than the median effective dose of paracetamol.^25,26^ This hypothesis was supported in this study as the difference in pain scores in both groups was insignificant, so the pain was not a considerable variable that it did not affect the results of the incidence of POD between groups.

Our findings can be explained by the biochemical properties of melatonin, which antagonizes the pathophysiological causes of delirium. Melatonin preserves the synchronization when circadian rhythms are endangered.^27^ It has an anti-oxidant action and thus decreases the cellular damage caused by the reactive oxygen species and limits the progression of neurodegenerative diseases.^28^ Melatonin has immune and anti-inflammatory effects by raising CD4 cells, decreasing CD8 cells, cytokine, and T-cell signaling control, and down-regulating the neuronal nitric oxide synthetase.^27^ It has a neuroprotective impact, as seen in animal models, by inhibiting excitotoxic injury, preventing ischemia-reperfusion injuries and improving regional cerebral blood flow.^29^

The effectiveness of melatonin supplementation in the reduction of POD can be based on the reduced plasma level of melatonin that may be found in the targeted population. Melatonin secretion decreases rapidly with age.^30^ POD has been linked to a considerable drop in melatonin levels following surgery relative to the preoperative levels in old adults.^31^ A reciprocal relationship exists between melatonin and cortisol levels. So, severe stress that is induced by trauma and surgery may lower plasma concentrations of postoperative melatonin.^32^

The circadian rhythm of melatonin secretion, through the suprachiasmatic nucleus regulation of daytime N-acetyltransferase, is adequately strong to inhibit daytime darkness from triggering melatonin secretion. As a consequence of GA, darkness was proved not to be associated with increased plasma melatonin.^33^ GA, conversely, has been associated with decreased melatonin levels in some trials.^34-35^ On the first postoperative day, surgical patients experience a decrease in plasma melatonin levels. However, most of the analyzed patients had a regular circadian melatonin secretion pattern by the second postoperative night, and this may explain the negligible difference between groups that began on the second postoperative day (day-1).^14,34^

Other analyses have demonstrated similar findings to this research.^36^ Artemiou et al^37^ stated that melatonin prophylaxis in heart surgery lowered the frequency of delirium from 20.8% in the control group to 8.4% in the melatonin group, with a significant intergroup difference. However, in our research, cardiac surgery has not been included as the pathogenesis of delirium is influenced by cardiopulmonary bypass, which is not the case in our investigation. Sultan^15^ tested melatonin role in the management of POD in 300 elderly patients after spinal anaesthesia for hip arthroplasty and recorded a substantial reduction in the occurrence of POD to 9.43% and an efficiency of melatonin to treat greater than half of POD patients in 3 postoperative days. However, the author did not study its effect under GA.

In contrast to our study, a multicenter, double-blind RCT rendered by De Jonghe et al^38^ did not notice a better consequence of melatonin to the frequency of POD following hip fracture than placebo. However, the author used a lower dose of melatonin (3 mg) than in our study.

The impact of exogenous melatonin on POD prevention was the concern of many researchers that meta-analyses were conducted to investigate this issue. A meta-analysis by Campbell et al^39^ concluded that prophylactic melatonin substantially lowered the frequency of delirium in surgical older people with an OR of 0.63 (95% CI 0.46-0.87; *P  = *.006; I^2^ = 72.1%). Han et al^18^ declared that in surgical populations, melatonin or its analog (ramelteon) decreased the prevalence of POD with an OR = 0.45, 95% CI 0.24‐0.84, *P * = .01. In another meta-analysis, done by Chen et al^13^ a reduction in the POD incidence was found in patients receiving melatonin (relative risk [RR] 0.41, 95% CI 0.15-1.13; *P * = .08) relative to the control group. Ng et al^40^ underwent a meta-analysis of melatonin impact on delirium occurrence in the hospitalized patients and observed a nonsignificant reduction in the prevalence in patients who were administered melatonin. The authors did not support or antagonize the usage of melatonin to prevent POD in these patients’ populations.

However, these meta-analyses either included medical patients or studies on cardiac surgeries. Besides, not all of them included geriatric patients > 65 years old or were concerned with the type of anaesthesia.

The findings of this study revealed a statistically higher sedation score, surgery duration, and BMI in patients having delirium (**delirium melatonin group)** than in patients who did not have delirium (**non-delirium melatonin**) within the melatonin group. Otherwise, no significant difference occurred as regards the other risk variables. Patient and anaesthetic factors, as well as the stress of the intervention, can affect the equilibrium between melatonin secretion and removal. Therefore, based on this equilibrium, perioperative plasma levels of melatonin may be elevated or lowered relative to the regular physiological daytime levels, which may clarify the existence of the delirium melatonin group.^41^

The study lacks the use of different melatonin doses; however, this effective dose was selected based on previous studies. This work depended, only, on the AMT score rather than other scores for evaluation of POD. Yet, the AMT score was proved by prior studies to be an effective simple score in the diagnosis of delirium. AMT scoring was done at a fixed time every day, so the day-night difference in the incidence of POD could not be evaluated. The study didn’t include cardiac surgeries to avoid the effect of cardiopulmonary bypass on the prevalence of POD. In this study, the authors did not use different intraoperative opioids, nerve blocks, or patient-controlled analgesia to control pain which should be considered in future studies. The study was designed not to follow patients for more than 3 days. However, this did not affect the significance of the results. Not all risk factors of POD were targeted in this study. Finally, ASA IV patients were not included. These limitations open a wide door for future researches on this topic.

## Conclusion

Perioperative melatonin administration could decrease the incidence of POD after orthopaedic trauma surgery under GA in geriatric patients. AMT scores were significantly higher in patients who were given melatonin in PACU and day [0]. It is concluded that routine prophylactic melatonin could be considered for surgeries on patients similar to this study population.

## Figures and Tables

**Table 1.** The AMT Questionnaire

**AMT Questionnaire**
1. Patient’s name
2. Time (to the closest hour)
3. Address to recall when ending the investigation
4. The current year
5. The name of the hospital
6. Recognizing 2 surrounding personnel (e.g., physician, nurse)
7. Patient’s birth date
8. Year of any famous event
9. Name of the present president
10. Count backward from 20 to 1
Each answer scored 1 point and the total score was calculated.

**Table 2.** Pain Assessment in Advanced Dementia (PAINAD) Scale

**Items**	Score = 0	Score = 1	Score = 2
Breathing	Normal	Sporadic labored breathingA brief period of hyperventilation	Noisy labored breathingProlonged duration of hyperventilationCheyne-Stokes respirations
Negative vocalization	None	Sporadic moans or groansLow degree of speech with a negative quality	Repetitive distressed calling outLoud moans or groansCrying
Facial expression	Smily or nonexpressive	SadFrightened	Grimacing
Body language	Calm	TenseDistressed	RigidFists clenchedKnees pulled upPulling or pushing away.
Consolability	No need to	Diverted or calmed by voice or touch	Cannot console

*Overall scores range from 0 to 10, with a higher score indicating more intense pain (0 = “no pain” to 10 = “severe pain”).

**Table 3.** Patients’ Characteristics in M and NM Groups

		**M group (no. 40)**		**NM group (no. 40)**		*P*
**Count**	**%**	**Count**	**%**
**Gender**	Male/Female	18/22	45/55	21/19	52.5/47.5	.230
**ASA**	**I/II/III**	4/28/8	10/70/20	5/26/9	12.5/65/22.5	.673
**Delirium incidence**	**Yes**	10	25	21	52.5	<.001
**No**	30	75	19	47.5
**Sedation score**	**1**	11	27.5	15	37.5	.280
**2**	29	72.5	25	62.5
**High perioperative transfusion needs**		6	15	4	10	.320
**Intraoperative fluctuation of pressure **		8	20	7	17.5	.770
**Hypoxia**		4	10	4	10	1

**Table 4.** Comparison of AMT Scores Between M and NM Groups

	**M group (no. 40)**	**NM group (no. 40)**	*P*
**Before surgery**			
Mean ± SD	9.32 ± 0.77	9.20 ± 0.70	.42
**at PACU**			
Mean ± SD	7.98 ± 2.36	6.4 ± 2.39	.001
** N (%) of POD**	7 (17.5%)	8 (20%)	.77
**Day 0**			
Mean ± SD	8.74 ± 0.96	7.16 ± 0.96	.04
** N (%) of POD**	2 (5%)	8 (20%)	.042
**Day 1**			
Mean ± SD	9.26 ± 0.77	8.9 ± 0.76	.23
** N (%) of POD**	0 (0%)	4 (10%)	.04
**Day 2**			
Mean ± SD	9.32 ± 0.73	9.1 ± 0.8	.34
** N (%) of POD**	1 (2.5%)	1 (2.5%)	1
**Day 3**			
Mean ± SD	9.32 ± 0.68	9.2 ± 0.67	.44
** N (%) of POD**	0 (0%)	0 (0%)	
**24 hours PAINAD scale**	3.6 ± 1.4	3.84 ± 1.7	.64

*Data are expressed as mean ± standard deviation (SD), Numbers (N.) and percent (%),; AMT, the Abbreviated Mental Test; PACU, postanaesthesia care unit; PAINAD, Pain Assessment in Advanced Dementia scale; *P* < .05, significant difference; *P* < .001, highly significant difference.

**Table 5.** Patients’ Characteristics of Delirium and Non-delirium Melatonin Group

	**Delirium melatonin**		**Non-delirium melatonin**		*P*
**Count**	**%**	**Count**	**%**
**Gender**					
** Male**	5	50	12	40	.631
** Female**	5	50	18	60
**ASA**					
** I**	1	10	3	10	.735
** II**	6	60	22	73.3	
** III**	3	30	5	16.7
**Sedation score**					
** 1**	5	50	6	20	.049
** 2**	5	50	24	80
**High perioperative transfusion needs**	3	30	3	10	.260
**Intraoperative fluctuation of pressure **	4	40	4	13.3	.100
**Hypoxia**	2	20	2	6	.066
	**Delirium melatonin**		**Non-delirium melatonin**		
**Mean**	**SD**	**Mean**	**SD**	*P* ** value**
**Age (years)**	70.33	4.33	68.47	3.50	.136
**BMI (kg m** **-2** **)**	29.83	5.2	24.9	3.9	.030
**Duration of surgery (minutes)**	113	25.4		22.2	.042

*Data are expressed as numbers, mean ± standard deviation (SD) and percent (%). BMI, body mass index; ASA, the American Society of Anesthesiologists; *P* < .05, significant difference; *P* < .001, highly significant difference.

**Table 6.** Comparison of AMT Scores Between the Delirium and Non-delirium Melatonin Groups

	**Delirium melatonin**		**Non-delirium melatonin**		*P* ** value**
**Mean**	**SD**	**Mean**	**SD**
**Before surgery**	9.17	0.83	9.37	0.75	.433
**at PACU**	4.00	1.3	8.4	1.47	< .001
**Day 0**	6.75	1.44	9.33	1.01	.020
**Day 1**	8.9	1.1	9.36	0.78	.376
**Day 2**	9.12	0.71	9.37	0.785	.487
**Day 3**	9.12	0.71	9.37	0.785	.487
**24 hours PAINAD scale**	3.8	1.41	3.42	1.24	.090

*Data are expressed as mean ± standard deviation (SD) and percent (%); AMT, the Abbreviated Mental Test; PACU, postanaesthesia care unit; PAINAD, Pain Assessment in Advanced Dementia scale, *P* value < .05, significant difference; *P* value < .001, highly significant difference.
